# Characterization of focal liver lesions using quantitative techniques: comparison of apparent diffusion coefficient values and T2 relaxation times

**DOI:** 10.1007/s00330-012-2519-x

**Published:** 2012-06-15

**Authors:** Andrzej Cieszanowski, Agnieszka Anysz-Grodzicka, Wojciech Szeszkowski, Bartosz Kaczynski, Edyta Maj, Barbara Gornicka, Mariusz Grodzicki, Ireneusz P. Grudzinski, Anna Stadnik, Marek Krawczyk, Olgierd Rowinski

**Affiliations:** 1 2nd Department of Clinical Radiology, Medical University of Warsaw, Ul. Banacha 1A, 02-097 Warsaw, Poland; 2 Department of Pathological Anatomy, Medical University of Warsaw, Warsaw, Poland; 3 Department of General, Transplantation and Liver Surgery, Medical University of Warsaw, Warsaw, Poland; 4 Department of Toxicology, Medical University of Warsaw, Warsaw, Poland

**Keywords:** Magnetic resonance, Echo-planar imaging, Diffusion-weighted imaging, Liver neoplasms, Quantitative analysis

## Abstract

**Objectives:**

To compare the efficacy of two quantitative methods for discrimination between benign and malignant focal liver lesions (FLLs): apparent diffusion coefficient (ADC) values and T2 relaxation times.

**Methods:**

Seventy-three patients with 215 confirmed FLLs (115 benign, 100 malignant) underwent 1.5-T MRI with respiratory-triggered single-shot SE DWI (*b* = 50, 400, 800) and dual-echo T2TSE (TR = 3,000 ms; TE1 = 84 ms; TE2 = 228 ms). ADC values and T2 relaxation times of FLLs were calculated. Sensitivity, specificity and accuracy of both techniques in diagnosing malignancy were assessed.

**Results:**

The mean ADC value of malignant tumours (1.07 × 10^−3^ mm^2^/s) was significantly lower (*P* < 0.05) than that of benign lesions (1.86 × 10^−3^ mm^2^/s ); however, with the use of the optimal cut-off value of 1.25 × 10^−3^ mm^2^/s, 20 false positive (FP) and 20 false negative (FN) diagnoses of malignancy were noted, generating 79 % sensitivity, 82.6 % specificity and 80.9 % accuracy. The mean T2 relaxation time of malignant tumours (64.4 ms) was significantly lower (*P* < 0.05) than that of benign lesions (476.1 ms). At the threshold of 107 ms 22 FP and 1 FN diagnoses were noted; the sensitivity was 99 %, specificity 80.9 % and accuracy 89.3 %.

**Conclusions:**

Quantitative analysis of T2 relaxation times yielded significantly higher sensitivity and accuracy in diagnosing malignant liver tumour than ADC values.

**Key Points:**

• *Diffusion-weighted magnetic resonance imaging is increasingly used for liver lesions.*

• *But ADC values demonstrated only moderate accuracy for differentiation of liver lesions.*

• *T2 relaxation times yielded higher accuracy in diagnosing malignant liver tumours.*

• *Both ADC and T2 values overlapped between focal nodular hyperplasia and malignant lesions.*

• *Nevertheless T2 liver mapping could be valuable for evaluating focal liver lesions.*

## Introduction

During the past decade magnetic resonance imaging (MRI) has emerged as the leading modality for the detection, characterisation and preoperative assessment of focal liver lesions (FLLs) [1–7]. Advances in MRI technology have allowed the implementation of several MR sequences, such as 3D T1-weighted fast spoiled gradient echo (GE) and diffusion-weighted imaging echo-planar imaging (DW EPI), for the routine application in abdominal imaging.

Researchers recently investigated the efficacy of DW imaging based on quantitative analysis of apparent diffusion coefficient (ADC) values of liver lesions to differentiate between benign and malignant lesions [8–21]. The results of most of them were promising, as they demonstrated statistically significant differences between higher mean ADC values of benign lesions and lower mean ADC values of malignant tumours [7, 9–13, 16, 18]. Since the diffusion coefficient is related to lesion cellularity and the size of extracellular space, some highly cellular benign lesions such as focal nodular hyperplasia (FNH) or hepatocellular adenoma (HCA) showed lower ADC values in the range of those of malignant lesions [14]. Moreover, in a number of abscesses diffusion was restricted because of cellular debris and exudates [22]. Conversely, some malignant lesions, mostly metastases, demonstrated high ADC values [12, 14].

Before the implementation of DW MR liver imaging, another quantitative method based on calculation of T2 relaxation times of FLLs was used for the characterisation of liver tumours by several investigators [23–33]. Analysis of T2 relaxation times of hepatic lesions enabled the discrimination of nonsolid and solid liver lesions. Whereas the first group contains almost exclusively benign lesions (e.g. cysts, haemangiomas, abscesses), the second encompasses the majority of malignant masses (e.g. metastases, hepatocellular carcinoma, cholangiocarcinoma), but also includes some benign tumours, such as FNH and HCA, which often exhibit T2 values similar to those of malignant neoplasms [32].

To our knowledge, there are no published studies comparing ADC values obtained with DW MR imaging and T2 relaxation times for the differentiation of liver lesions. Thus, the purpose of this study was to compare the accuracy of two quantitative techniques for the characterisation of focal liver lesions: evaluation of ADC values based on the SE EPI sequence and assessment of T2 relaxation times derived from the double-echo TSE sequence.

## Materials and methods

### Study population

From May 2008 to January 2010, 125 patients underwent MR imaging of the liver at our institution. The MRI studies were conducted according to our routine liver protocol, which included respiratory-triggered diffusion-weighted single-shot echo-planar imaging (DW-SS-EPI) and dual-echo turbo spin-echo sequence. Fifty-two patients were excluded from our analysis due to (1) lack of sufficient data to confirm the nature of the lesions, (2) no focal liver lesion detected on MR imaging or (3) small size of FLLs detected on MR imaging (maximum diameter less than 5 mm). Thus, the study group comprised 73 patients (34 male, 39 female) with a mean age of 54.2 years (age range 18–84 years). Six patients had liver cirrhosis. In one patient with hepatocellular carcinoma there was no evidence of hepatic cirrhosis. One patient underwent right hemihepatectomy for treatment of liver metastases before MR imaging; another underwent hepatic segmentectomy due to HCC before MR. Three patients had previously been treated with chemotherapy, although not within the year before MR.

Hepatic lesions were solitary in 19 patients and multiple in 54 patients. In 24 patients only malignant liver lesions were detected, in 7 patients both malignant and benign lesions were diagnosed and the remaining 42 patients had only benign lesions, although 8 of them had more than one type of benign lesion. In patients with multiple lesions, a maximum of 5 lesions per patient were selected for analysis (including lesions which were largest, most conspicuous and easiest to localise).

A total of 215 hepatic lesions were evaluated (115 benign, 100 malignant) comprising 47 haemangiomas, 42 cysts, 13 FNHs, 12 abscesses, 1 biliary cystadenoma, 65 metastases, 19 hepatocellular carcinomas (HCCs), 13 haemangioendotheliomas, 2 peripheral cholangiocarcinomas (CCAs) and 1 biliary cystadenocarcinoma. The primary sites of metastatic lesions included colorectal carcinoma (*n* = 20), neuroendocrine carcinoma (*n* = 20), pancreatic adenocarcinoma (*n* = 16), gallbladder carcinoma (*n* = 5), lung cancer (*n* = 3) and sarcoma (*n* = 1).

In 36 of 73 patients (49 %) the reference standard for the diagnosis was histopathologic proof obtained intra-operatively in 12 patients (10 patients with malignant tumours and 2 patients with benign lesions: 1 abscess and 1 biliary cystadenoma) and from biopsy in 24 patients (21 patients with malignant masses and 3 patients with benign lesions: 1 haemangioma, 1 FNH, 1 abscess). In the remaining 37 patients no histological proof was obtained and the final diagnosis was based on results of previous imaging studies and the follow-up with a minimum of 6 months’ observation period (US, CT, MRI), laboratory tests and clinical data.

### MR imaging

Magnetic resonance imaging was performed on a 1.5-T system (Magnetom Avanto, Siemens Medical Solutions, Erlangen, Germany), using a phased-array multicoil system (12 elements).

Analysed breath-hold dual-echo T2-weighted TSE images and DW-SS-EPI were part of our routine liver imaging protocol, which consisted of the respiratory-triggered T2-weighted TSE sequence with fat saturation, the breath-hold in- and out-of-phase spoiled gradient dual-echo sequence, and a dynamic contrast-enhanced 3D gradient-echo volumetric interpolated breath-hold examination (VIBE) sequence, performed during arterial, portal venous, equilibrium and delayed phases and in hepato-biliary phase (after injection of gadobenate dimeglumine).

Diffusion-weighted images were acquired using the SE single-shot echo-planar sequence in the axial plane with respiratory triggering. Integrated parallel imaging technique (iPAT) with generalised autocalibrating partially parallel acquisition (GRAPPA) and an acceleration factor of 2 was applied. The other parameters were as follows: repetition time (TR) 1,700 ms, TE 90 ms, flip angle 90°, EPI factor 120, slice thickness 6 mm, 120 × 192 matrix, 2 acquisitions, field of view 344 mm, bandwidth 1,736 Hz/pixel, spectral fat suppression. Diffusion gradients were applied in three orthogonal directions separately with three increasing *b* values of 50, 400 and 800 s/mm^2^.

The breath-hold dual-echo TSE sequence was performed in the axial plane with repetition time (TR) 1,800 ms, first effective TE (TEeff) 84 ms, second TEeff 228 ms, flip angle 150°, turbo factor 29, slice thickness 6 mm, 207 × 256 matrix, 1 acquisition, acceleration factor of 2, field of view 340 mm, bandwidth 260 Hz/pixel.

### Image analysis

ADC measurements were performed on a commercial workstation (Leonardo, Siemens Medical Solution, Erlangen, Germany) by drawing regions of interest (ROIs) on DWI images, which provided the best delineation of analysed liver lesions. The ROI included the largest possible part of the lesion, avoiding partial volume effects, areas of necrosis, blood vessels and artefacts. Then the ROI was copied and pasted from the DWI image to the corresponding ADC map and the measurement on ADC map was recorded. The ADC was measured twice for each lesion and the two measurements were averaged.

ADC values were calculated by mono-exponential regression with following formula: *S* = *S*_0_ exp(−*b* ADC), where *S* is the signal intensity after application of the diffusion gradient and *S*_0_ is the signal intensity at *b* = 0 s/mm^2^. Three *b* values (50, 400, 800 s/mm^2^) were applied for ADC calculation.

On dual-echo T2-weighted TSE images signal intensities (SI) of all analysed lesions were measured, separately for each echo time (TE1 = 84 ms, TE2 = 228 ms). Two measurements of SI of each lesion were performed and the mean values were used for calculation of the T2 relaxation time. If the lesion consisted of solid and liquid parts, measurements (ROIs) were confined to its solid component, just as in ADC measurements.

Assuming that TR is much greater than T1, the standard equation for SE signal intensity may be simplified to SI = *K* exp^−(TE/T2)^, where *K* is a machine-dependent constant [3]. Therefore, the natural logarithm of signal intensity on SE image is linearly related to TE with a slope of −1/T2 [8].

T2 relaxation times of liver lesions were calculated according to the following formula:

$$ {\text{T2 (ms) = (TE2 }} - {\text{ TE1)}}/{\text{(ln SI1 }} - {\text{ ln SI2),}} $$

where TE1 is the first echo time (84 ms), TE2 is the second echo time (228 ms), ln SI1 and ln SI2 are the natural logarithms of the measured SI for TE1 and TE2, respectively.

### Statistical analysis

The statistical analysis was performed using Statistica software (version 10.0).

The Mann–Whitney *U* test was applied to assess statistically significant differences between the mean ADC values and between mean T2 relaxation times of benign and malignant focal liver lesions. This test was also used to assess statistically significant differences between lesions’ sizes in selected groups.

The χ^2^ test was used to compare sensitivities, specificities, accuracies, positive predictive values (PPV) and negative predictive values (NPV) of both quantitative techniques implemented for differentiation of FLLs. A *P* value of less than 0.05 was considered significant.

A receiver operating characteristic (ROC) curve analysis was implemented to define ADC and T2 cut-off values for the optimal differentiation of benign and malignant liver lesions.

The accuracy of each MR technique (ADC versus T2 relaxation times) was determined by calculating the area under the ROC curve (AUC). The differences between ROC curves were tested for significance.

## Results

Mean ADC values and mean T2 relaxation times of analysed benign and malignant hepatic lesions are shown in Table 1.

**Table 1:** Mean ADC values and mean T2 relaxation times of benign and malignant liver lesions and corresponding 95 % CI

Type of lesion (number)	Mean ADC (×10^−3^ mm^2^/s) 95 % CI	Mean T2 (ms) 95 % CI
Benign lesions (*n* = 115)		
Haemangioma (*n* = 47)	1.55 (1.465–1.641)	124.3 (116.74–131.92)
Cyst (*n* = 42)	2.45 (1.282–2.621)	1,007 (821.18–1,192.78)
FNH (*n* = 13)	1.18 (0.994–1.36)	62.8 (54.8–70.77)
Abscess (*n* = 12)	1.5 (1.147–1.856)	406.8 (133.86–679.82)
Cystadenoma (*n* = 1)	3.3	459
Malignant lesions (*n* = 100)		
Metastasis (*n* = 65)	1.05 (0.934–1.169)	65.3 (61.68–68.92)
HCC (*n* = 19)	0.94 (0.876–1.0)	59.1 (55.42–62.77)
Haemangioendothelioma (*n* = 13)	1.3 (1.171–1.437)	64.9 (56.64–73.16)
Cholangiocarcinoma (*n* = 2)	0.89	55.7
Cystadenocarcinoma (*n* = 1)	2.2	117.5

**Fig. 1:**
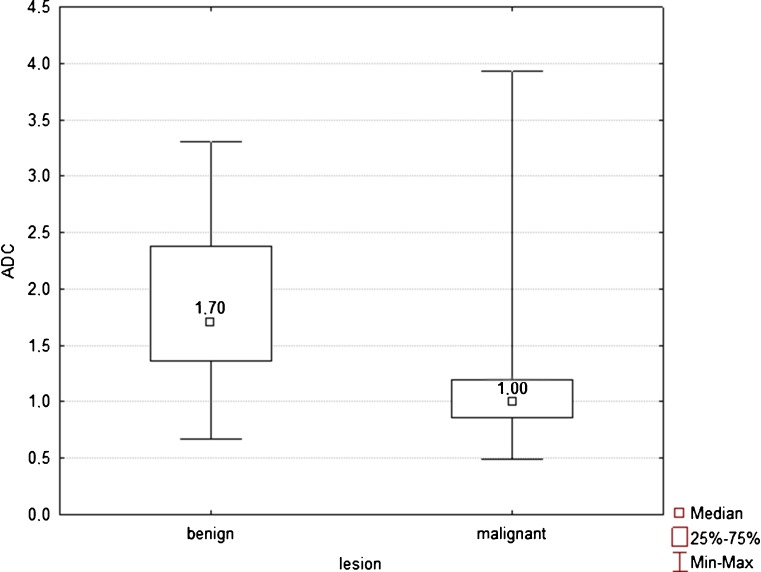
Box plots of ADC values of 115 benign and 100 malignant liver lesions show that despite ADC values of benign lesions being significantly higher than those of malignant tumours (*P* < 0.001), ADC values of both lesion types considerably overlapped. Median is shown as a *small box* inside each bar

The mean ADC value of malignant FLLs was 1.07 × 10^−3^ mm^2^/s, ranging from 0.74 × 10^−3^ to 2.2 × 10^−3^ mm^2^/s, whereas the mean ADC value of benign FLLs was 1.86 × 10^−3^ mm^2^/s, ranging from 0.67 × 10^−3^ to 3.22 × 10^−3^ mm^2^/s (Fig. 1). The difference between mean ADC values of malignant and benign FLLs was statistically significant (*P* < 0.001). The calculated area under the ROC curve for diagnosing malignant lesion was 0.874 (95 % CI 0.823, 0.962), with a sensitivity of 79 % and a specificity of 82.6 %, using a cut-off ADC value of 1.25 × 10^−3^ mm^2^/s.

**Fig. 2:**
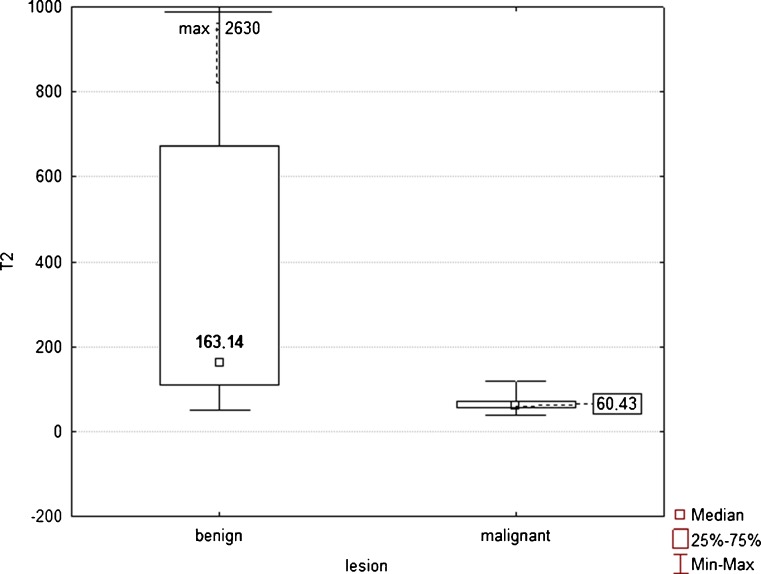
Box plots of T2 relaxation times of 115 benign and 100 malignant liver lesions show that T2 times of benign lesions were significantly higher than those of malignant tumours (*p* < 0.001), although there is some overlap. Median is shown as a *small box* inside (benign lesions) or outside (malignant lesions) bars. Nineteen benign lesions (18 cysts and 1 abscess) with T2 relaxation times above 1,000 ms were excluded from the plot for the sake of better visualisation

**Fig. 3:**
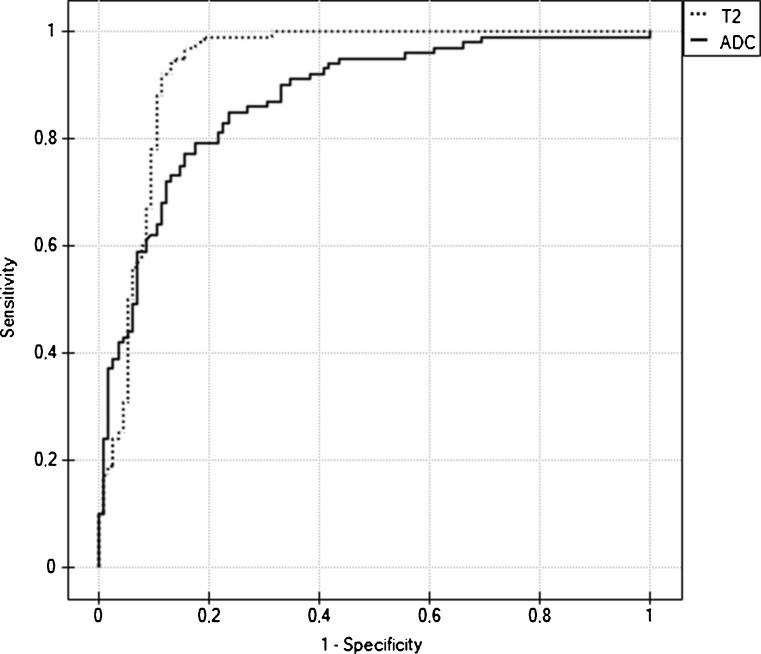
ROC curves for ADC values (*line*) and T2 relaxation times (*dots*) show that T2 times were more effective than ADC values in classification of focal liver lesions. The area under the ROC curve for T2 times (0.932; 95 % CI 0.891, 0.962) was significantly larger than the area under the ROC curve for ADC values (0.874; 95 % CI 0.823, 0.962)

The mean T2 relaxation time of malignant FLLs was lower than that of benign FLLs: 64.4 ms (range 45.83–117.49 ms) vs. 476.06 ms (range 49.87–2,630.82 ms) and the difference was statistically significant (*P* < 0.001; Fig. 2). The area under the ROC curve for diagnosing malignancy was 0.932 (95 % CI 0.891, 0.962), with sensitivity of 99 % and specificity of 80.9 %, using a threshold of 107 ms. The area under the ROC curve for T2 times was significantly larger (*P* < 0.001) than the area under the ROC curve for ADC values (Fig. 3).

The sensitivities, specificities, accuracies, PPV and NPV for both quantitative techniques (ADC values and T2 relaxation times) at optimal threshold values are summarised in Table 2.

**Table 2:** Sensitivities, specificities, PPV, NPV and accuracies for diagnosing malignancy using optimal cut-off ADC values and T2 relaxation times

	ADC values	T2 times
Cut-off values	1.25 (×10^−3^ mm^2^/s)	107 (ms)
Sensitivity (%)	79	99
Specificity (%)	82.6	80.9
Accuracy (%)	80.9	89.3
PPV (%)	79.8	81.8
NPV (%)	81.9	98.9

Analysis of T2 relaxation times yielded significantly higher sensitivity (*P* < 0.001), accuracy (*P* = 0.015) and NPV (*P* < 0.001) for diagnosing a malignant lesion than the use of ADC values, whereas the differences in specificity (*P* = 0.64) and PPV (*P* = 0.59) were not statistically significant.

**Fig. 4:**
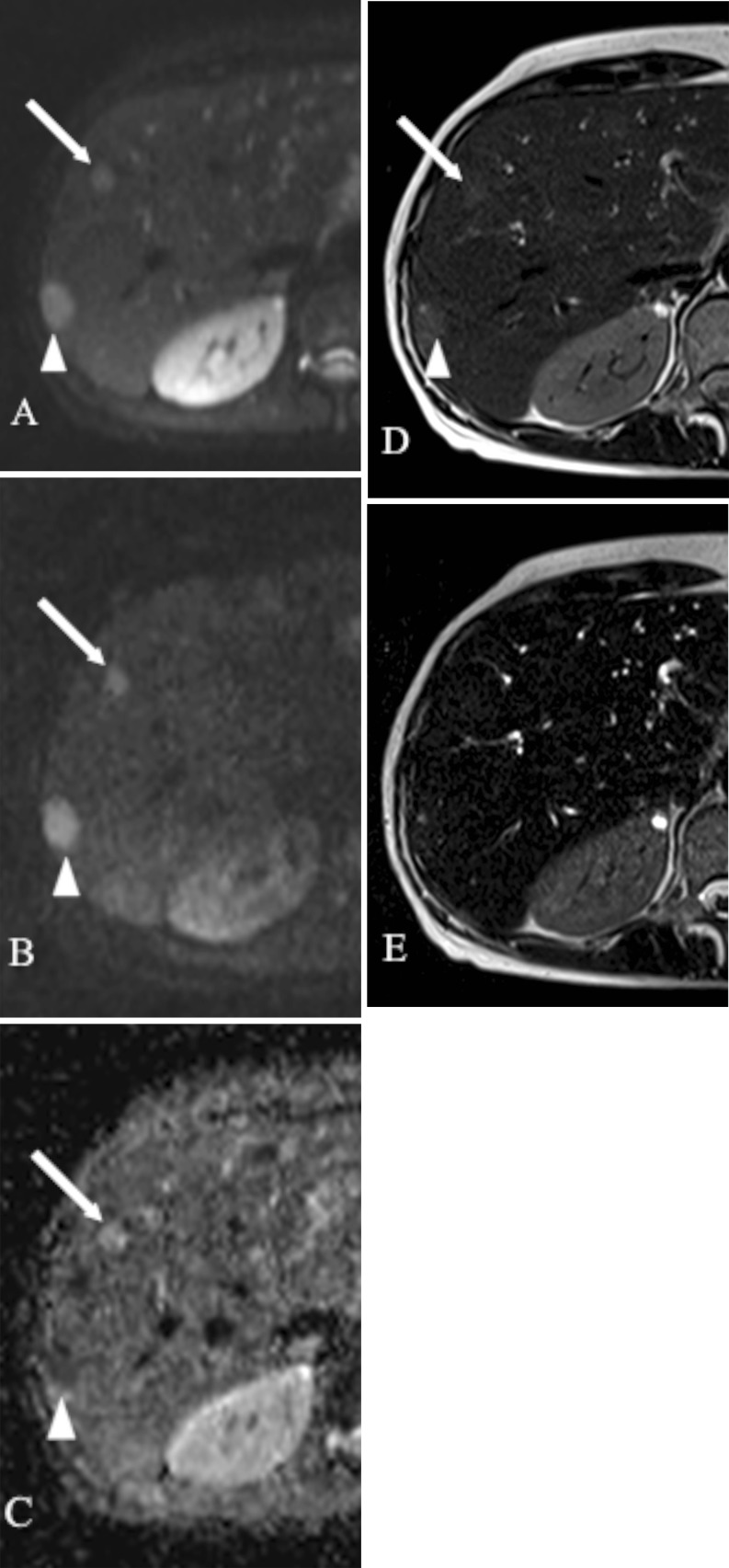
MR images obtained in a 51-year-old woman with liver metastases from neuroendocrine cancer. **a** *b* = 50 s/mm^2^ DW SS EPI image. **b** *b* = 800 s/mm^2^ DW SS EPI image. **c** Corresponding ADC map. Double echo TSE images: **d** TR/TE: 3,000/84 ms, **e** TR/TE 3,000/228 ms. The first metastatic lesion (*arrow*) displaying increased signal intensity on *b* = 50 and *b* = 800 images and on ADC map had an ADC value of 1.43 × 10^−3^ mm^2^/s (false negative diagnosis of malignancy). The second lesion (*arrowhead*) shows increased signal intensity on *b* = 50 and *b* = 800 images and decreased signal on ADC map consistent with restricted diffusion (ADC value = 1.24 × 10^−3^ mm^2^/s). Both lesions had T2 relaxation times in the range of those of a malignant lesion (58.9 ms and 98.6 ms)

**Fig. 5:**
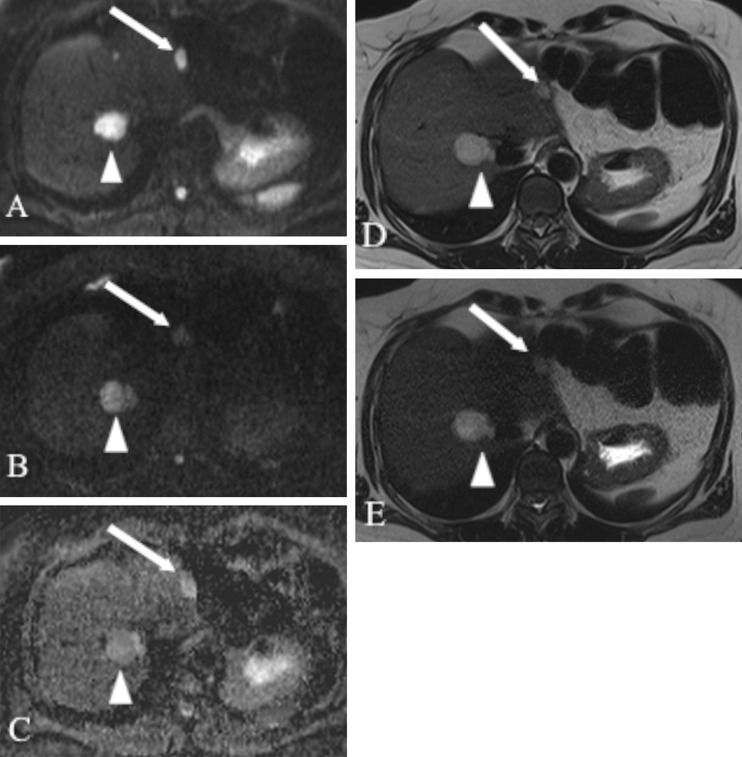
MR images obtained in a 53-year-old woman with hepatic haemangiomas. **a** *b* = 50 s/mm^2^ DW SS EPI image. **b** *b* = 800 s/mm^2^ DW SS EPI image. **c** Corresponding ADC map. Double echo TSE images: **d** TR/TE 3,000/84 ms, **e** TR/TE 3,000/228 ms. Both haemangiomas show increased signal intensity on *b* = 50 and *b* = 800 images and on ADC map; however, in the case of the haemangioma located in the left liver lobe (*arrow*), the decrease in signal intensity on *b* = 800 image and hyperintensity on ADC map are more pronounced (ADC value = 1.75 × 10^−3^ mm^2^/s—true negative case) than in the second haemangioma (*arrowhead*) located in the right liver lobe (ADC value of 1.23 × 10^−3^ mm^2^/s—false positive diagnosis of malignancy). T2 time of the haemangioma in left liver lobe (*arrow*) is in the range of those of malignancies (80.9 ms), whereas T2 time of second haemangioma (125.5 ms) is in the range of those of benign lesions

**Fig. 6:**
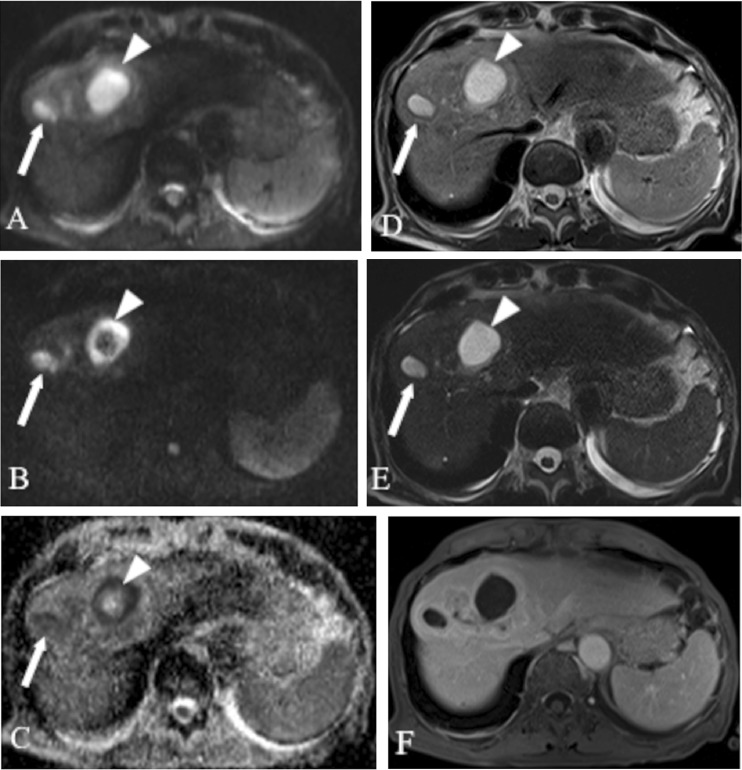
MR images obtained in an 83-year-old man with hepatic abscesses located in segment VIII. **a** *b* = 50 s/mm^2^ DW SS EPI image. **b** *b* = 800 s/mm^2^ DW SS EPI image. **c** Corresponding ADC map. Double echo TSE images: **d** TR/TE 3,000/84 ms, **e** TR/TE 3,000/228 ms. On DW images (*b *= 50, *b* = 800) the cavity of the first abscess (*arrow*) displays high signal intensity, which markedly decreases on ADC map, representing restricted diffusion (ADC value of 0.94 × 10^−3^ mm^2^/s—false positive diagnosis of malignancy). The cavity of the second abscess (*arrowhead*) shows increased signal on *b* = 50 DW image, mixed on *b* = 800 DW image and mostly increased on ADC map (ADC value of 1.27 × 10^−3^ mm^2^/s in the range of those of benign lesions). On axial T2-weighted images obtained with TE of 84 ms (**d**) and 228 ms (**e**) increased signal in noted in both cavities corresponding to relative long T2 times of 293 ms and 238 ms, typical for benign lesions. Abscesses demonstrate typical peripheral capsular enhancement on portal phase 3D GRE axial image (**f**)

With the use of ADC values 20 false positive diagnoses of malignant lesions (9 FNHs, 6 haemangiomas, 3 abscesses, 2 cysts) and 21 false negative cases (13 metastases, 7 haemangioendotheliomas, 1 cystadenocarcinoma) were noted (Figs. 4, 5 and 6). Therefore, a total of 41 FLLs were misclassified using ADC quantifications. The primary sites of 13 misclassified metastatic lesions included neuroendocrine tumour (*n* = 5; Fig. 4), pancreatic adenocarcinoma (*n* = 4), colorectal carcinoma (*n* = 3) and sarcoma (*n* = 1).

The implementation of T2 calculations resulted in 22 false positive diagnoses of malignant FLL (13 FNHs, 8 haemangiomas, 1 cyst) and only one false negative case (biliary cystadenocarcinoma). No metastatic lesion or HCC was misclassified with the use of this technique. In total, quantitative analysis of T2 relaxation times led to 23 hepatic lesion misclassifications, including all examined FNHs. The mean size of 8 haemangiomas falsely diagnosed as malignant (12.3 mm) was significantly smaller (*P* < 0.05) than the mean size of all studied haemangiomas (19.8 mm). Four out of 8 misclassified haemangiomas were smaller or equal to 8 mm, whereas the remaining lesions ranged from 13 to 21 mm (Fig. 5).

## Discussion

Several groups of researchers have published promising results regarding the implementation of DW MR imaging for the detection [2, 3, 7, 15, 34–40] and characterisation of focal liver lesions [7, 10–13, 16, 17]. Some of these publications have suggested that DW MR images and ADC maps may be useful in the differentiation of benign and malignant lesions [7, 10–13, 16, 18].

Given the limitations of visual assessment, quantitative analysis based on ADC values of FLLs has been proposed for the discrimination of benign from malignant lesions [7–16, 18–21]. However, investigators using ADC quantification have reported varying sensitivities (range 74–100 %) and different specificities (range 77–100 %) in diagnosing of malignant lesions [9, 10, 12–14] . Moreover, significant differences in ADC cut-offs ranging from 1.47 × 10^−3^ to 1.63 × 10^−3^ mm^2^/s have been proposed for the optimal differentiation of benign and malignant lesions [9, 10, 12–14]. In the current study, the sensitivity and specificity for the detection of malignant lesions were in the range of those of previous studies (79 % and 82.6 %, respectively), although the ADC cut-off was significantly lower (1.25 × 10^−3^ mm^2^/s). There are several possible reasons explaining this difference, including the use of different hardware, absence of standardization of image acquisition (choice of different *b* values), various methods for ADC calculation and different patient populations.

The choice of *b* values (number, range, the first *b* value) and the method for ADC calculation (mono-exponential versus bi-exponential model) have important implications for the calculated ADC values. For instance, application of a two-point mono-exponential regression with low *b* values (including *b* = 0 s/mm^2^) results in the overestimation of ADC (higher obtained ADC values) owing to the incorporated effect of perfusion [6, 41, 42]. The ADC calculated with higher *b* values (including the first *b* value of at least 50 s/mm^2^) is lower and probably more reliable [6, 42]. The DW technique used by us comprised multiple and higher *b* values than in the majority of prior studies and omitted the lowest *b* value of 0, leading to some decrease of the perfusion effect. Such a choice of parameters resulted in lower mean ADC values being obtained for liver lesions and a lower ADC cut-off for differentiation of FLLs.

The patient population also has important implications for the performance of ADC quantification in FLL characterisation. The best results were obtained by investigators who studied patients with no or a small number of solid benign tumours (FNH, HCA) and abscesses, which often have ADC values in the range of those of malignant lesions [12, 13]. Gourtsoyianni et al. studied a group of patients with 15 solid malignant tumours (13 metastases, 2 HCCs) and 22 non-solid benign lesions (15 cysts, 7 haemangiomas) obtaining the highest possible sensitivity and specificity (100 %) for diagnosing malignancy [13]. No solid benign lesions, such as FNH or HCA, were included in their study population, decreasing the probability of false positive diagnosis of malignant lesions. In the group of 204 liver lesions studied by Bruegel et al. only 4 FNHs were noted, representing less than 3.6 % of 111 analysed benign lesions [12]. These authors reported high sensitivity and specificity for diagnosing malignant tumour (90 % and 86 %, respectively) [12]. Less promising but probably more realistic results were obtained by Parikh et al. (74 % sensitivity, 77 % specificity), who analysed 211 liver lesions. In this group, the number and percentage of solid benign lesions (5 adenomas and 4 FNHs, 12 %) were higher and could influence the diagnostic performance of ADC values [14]. In our study group solid benign lesions (13 FNHs) constituted 11.3 % of all benign lesions, a percentage similar to that in Parikh et al.’s study. ADC values of 9 of 13 FNHs were in the range of those of malignant tumours (false positive diagnoses), leading, along with 3 abscesses and 6 haemangiomas, to a decrease in specificity (82.6 %).

In the current study we attempted to compare the performance of DW MRI with T2-weighted TSE imaging for the discrimination between benign and malignant lesions. Previously such comparisons were performed, but only by applying qualitative, visual assessment of FLLs on T2-weighted images [14, 15]. As reported by Fenlon et al., who compared quantitative and qualitative analyses of FLLs on T2-weighted SE images, quantitative evaluation of T2 relaxation times allowed more accurate and confident differentiation between benign and malignant lesions [31]. To the best of our knowledge, this study is the first attempt to compare two available quantitative methods of FLL characterisation: calculation of ADC values and T2 relaxation times.

In the past T2 quantifications were successfully applied by several groups using different techniques of image acquisition, including conventional SE, moderately T2-weighted TSE, heavily T2-weighted TSE and T2 EPI sequences with a varied number of echoes utilized for T2 calculations [23–33]. Goldberg et al., who derived T2 calculations from echo-planar sequences with four different echo times, obtained 100 % accuracy in the differentiation between solid and non-solid lesions [26]. McFarland et al., using a dual-echo heavily T2-weighted SE sequence, reported 100 % sensitivity and 92 % specificity for the discrimination of haemangiomas and malignant tumours [27]. Cieszanowski et al. implemented a dual-echo moderately T2-weighted sequence, achieving 98 % sensitivity and 96 % specificity, for diagnosing solid lesions [32].

In our comparative study of two quantitative techniques, the sensitivity for the diagnosis of malignant lesions was significantly higher with the use of T2 values (99 %) than with ADC values (79 %). Implementation of T2 times led to only a single false negative diagnosis of malignancy (cystadenocarcinoma), whereas ADC quantification resulted in 21 such cases (13 metastases, 7 haemangioendotheliomas, 1 cystadenocarcinoma). Slightly more numerous false positive diagnoses of malignant lesions were noted with analysis of T2 relaxation times than with ADC values (22 vs. 20) leading to better specificity of ADC quantification (82.6 % vs. 80.9 %). Moreover, 13 FNHs (all in our study population), which accounted for most of the 23 misclassified hepatic lesions by T2 quantifications, had typical appearance on contrast-enhanced images. Therefore, the combined evaluation of all obtained MR images would lead to the correct diagnosis in these cases.

Beside FNHs, the majority of remaining false positive cases of malignancy for T2 calculations were 8 haemangiomas (Fig. 5). Their mean size of 12.3 mm was considerably smaller than the mean size of all studied haemangiomas (19.8 mm). Thus, it is possible that the decrease of T2 relaxation times in some of these cases could result from the volume averaging effect.

Several limitations of this study should be mentioned. Firstly, we applied only three *b* values (50, 400, 800 s/mm^2^); therefore, calculated ACD values are approximate. The inclusion of more *b* values would probably lead to more accurate ADC calculation, however at the expense of prolonged examination time. Secondly, mono-exponential regression using three *b* values (50, 400, 800 s/mm^2^) was implemented for ADC calculations. The ADC value depends not only on diffusion but also incorporates the effect of perfusion and signal attenuation is bi-exponential with DW imaging. We cannot exclude that application of a bi-exponential model using more *b* values for ADC calculation would result in obtaining more reliable ADC values. Thirdly, we implemented only two echoes in the T2-weighted TSE sequence. Inclusion of more echoes would probably lead to more accurate estimation of T2 relaxation times of FLLs, but at the expense of extended imaging time. Fourthly, the composition of our study population did not fully represent the typical spectrum of liver lesions, including 13 haemangioendotheliomas and lacking adenomas. Finally, histopathological proof was not available in 37 of 73 patients (51 %), although it was obtained in all patients with malignant lesions.

The results of our study confirm that the DWI technique with ADC quantification has potential in the differentiation of FLLs. However, in this first published comparison of two quantitative techniques (ADC quantification vs. T2 times calculation) for characterisation of FLLs, T2 values yielded significantly higher sensitivity for diagnosing malignant lesions than ADC values. It is at variance with the results of previous publications comparing DW MRI with T2-weighted TSE imaging, although in these studies only qualitative assessment of T2-weighted images was employed [14, 15].

Compared with assessment of ADC values on ADC maps, generated automatically on a commercial workstation, analysis of T2 times of FLLs was not that simple and required separate, manual drawing of ROIs on images acquired with different TEs and then transferring all data to a PC to obtain T2 values using a commercial calculation sheet (Excel, Microsoft). In our opinion, inclusion of multiple-echo T2-weighted TSE sequences in MR protocols dedicated for liver imaging, along with automatically generated T2 maps on commercial MR consoles (similarly to ADC maps or T2 mapping used for assessment of articular cartilage), should be considered by MR vendors. We presume that it would provide important quantitative data, helpful in the differentiation of FLLs.

Despite the usefulness of T2 and ADC quantification for the differentiation of FLLs, some pitfalls of these techniques must be kept in mind. There is substantial overlap between highly cellular, solid benign lesions, such as FNH, HCA and malignant tumours (HCC, metastases) on both DW and T2-weighted TSE images. Moreover, some abscesses, due to cellular debris and exudates, may also display restricted diffusion. Therefore, DW and T2-weighted images should be used as complementary methods for the characterisation of FLLs, in conjunction with clinical context and pre- and post-contrast T1-weighted scans, including dynamic and hepatobiliary phase images. In our material the most important advantage of T2 quantification over ADC values was the more confident characterisation of metastatic lesions, abscesses and haemangioendotheliomas.

In conclusion, comparison of the two quantitative methods used for the characterisation of FLLs demonstrated significantly higher sensitivity and accuracy of T2 relaxation times than ADC values (99 % and 89.3 % versus 79 % and 80.9 %, respectively) for diagnosing hepatic malignancy. Inclusion of multiple-echo T2-weighted TSE sequences in MRI liver protocols together with T2 mapping of the liver on MR consoles may be an additional valuable tool facilitating the discrimination of malignant and benign hepatic lesions.
